# Effects of predisposing, reinforcing and enabling factors on self-care behaviors of the patients with diabetes mellitus in the Minoodasht city, Iran

**DOI:** 10.1186/s40200-015-0139-0

**Published:** 2015-04-14

**Authors:** Mahboobeh Borhani, Babak Rastgarimehr, Zahra Shafieyan, Morteza Mansourian, Seyed Mojtaba Hoseini, Seyed Masoud Arzaghi, Mostafa Qorbani, Aziz Rezapoor, Hamid Asayesh, Abdurrahman Charkazi, Hossein Ansari

**Affiliations:** Health Education and Promotion Department, Tehran University of Medical Sciences, Tehran, Iran; Abadan School of Medical Sciences, Abadan, Iran; Student Research Committee, Ilam University of Medical Sciences, Ilam, Iran; Public Health Department, Ilam University of Medical Sciences, Ilam, Iran; Phd Student in Exercise Physiology, Mazandaran University of Physical Education and Sport Sciences, Babolsar, Iran; Elderly Health Research Center, Endocrinology and Metabolism Population Science Institute, Tehran University of Medical Sciences, Tehran, Iran; Departments of Community Medicine, School of Medicine, Alborz University of Medical Sciences, Karaj, Iran; Non-Communicable Diseases Research Center, Endocrinology and Metabolism Research Institute, Tehran University of Medical Sciences, Tehran, Iran; Department of Health Economics, School of Health Management and Information Sciences and Health Management & Economics Research Center, Iran University of Medical Sciences, Tehran, Iran; Department of Medical Emergencies, Qom University of Medical Sciences, Qom, Iran; Department of Public Health, Golestan University of Medical Sciences, Gorgan, Iran; Health Promotion Research Center, Zahedan University of Medical Sciences, Zahedan, Iran

**Keywords:** Predisposing factors, Reinforcing factors, Enabling factors, Self-care behaviors, Diabetes mellitus

## Abstract

**Background:**

To control diabetes mellitus (DM) it is necessary to make overall changes in the life style of the patients. The aim of this study was to determine the effects of predisposing, reinforcing and enabling factors on self-care behaviors of the patients with DM in the Minoodasht city, Iran in 2012.

**Methods:**

In this quasi-experimental study, 78 people with DM were selected by convenience sampling method. In the first stage of study, the educational program was compiled and executed on six information sessions. To present the informative content, a video projector and different lecturing methods including questions and answers, dynamic group discussion and different educational materials such as pamphlets and CDs were employed. After one month, the efficiency of the educational program was determined by using the same questioner. Data were analyzed using paired sample *T*-test and McNemar test.

**Results:**

The mean age of participants was 49 (SD: 3.27.) years old, 87.2% were married, and 19.2% were illiterate. The results showed that the enabling factors like adopting to go on a diet and the educational classes facilitated by the staff had significant effects on health care behavior of the patients. Furthermore 69.2% of the participants adopted to go on a diet before the educational sessions; that figure increased to 94.9% after the educational sessions. According to the results the mean scores for the knowledge, attitude, and behavior, reinforcement factors and enabling factors increased significantly after of the educational intervention (*p-* value >0.001).

**Conclusion:**

Predisposing, enabling and reinforcement factors affected in taking self-care behavior in the patient with DM.

## Introduction

According to the report by World Health organization (WHO), the number of patients with DM would double in the next 25 years; resulting in an increase from 171 million people on the year 2000 to potentially 366 million people by year 2030 [1]. Every 10 seconds DM causes one death event. At the same time, 2 people are afflicted with the disease. International Federation of Diabetes Mellitus declared that 7 million people over the world are added to the population of patients with DM every year. In the near future, more than 350 million people will be suffering from DM whom is mainly Asians [2]. As the number of patients with DM is increasing in the world, the increase in the healthcare budget is inevitable, too. Without primary prevention the disease epidemics would continue growing even worse. It is believed that worldwide DM would become the main cause of morbidity and mortality in the next 25 years [1].

Diabetes mellitus (DM) is one of the common chronic metabolic illnesses and a major health problem that needs constant monitoring [3]. Furthermore, complications of DM are among the principle causes of mortality and morbidity in the world [4,5] and also in the Iran burden of DM and its complications is high [6]. DM is a serious, prevalent and costly disease [7]. Although, there is the possibility to control DM, but nearly 371 million people are suffering from this disease [8]. DM includes some groups of prevalent metabolic disorders which are common in hyperglycemic phonotype. In the USA, DM is the main cause of the acute and chronic renal failure, non-traumatic lower limb amputation and blindness in adult population [9]. As DM is spreading, it is estimated that this disease would continue to be one of the major causes of mortality in the world [10]. Being a chronic illness, to be able to control DM it is necessary to make overall changes in the life style of the patients. It is estimated that patients with DM can do self-monitoring in 95% of the cases [1].

It is easy to overcome many of the complications of DM or to postpone their occurrence by insulin control; and by providing a context for preventative measures such as early diagnosis, intervention, and implementing therapeutic treatments. Although, there is a good knowledge about the advantages of tenuous Insulin control and preventative measures, recent studies have shown that many individuals with DM have not received suitable monitoring care. This problem arises from differences in theory, education and principle interceptors [11]. Generally, people with DM have a lower health status and must pay a higher price for their therapeutic treatments.

In Iran, 4 million people are diagnosed with DM and this population increases by 120 thousand people every year. Although, it is evident that DM is the most common cause of disabilities such as physical, mental and psychological illnesses but more than half of the patients affected by DM are not aware of their disease [2].

According to the research studies, self-care behavior, healthy life style with a educational intervention program in a six years period lead to a decrease of almost two-thirds of the patients suffering from DM. These results, also, indicate that these methods are adequate effective measures [12]. The patients believe that it is difficult to carry out recommendations on healthy life style choices and to attend the healthcare screening session or to consume therapeutic medication [13]. As, diabetes is a chronic disease and entails a long process; it seems that one important guideline to improve the patients’ quality of life is to use an instructional model [14]. One of the models which are used in different studies for diagnosing, implementing treatments, and preventing the disease, which is also used in the present study, is the **p**redisposing, **r**einforcing and **e**nabling **c**onstructs in **e**ducational **d**iagnosis and **e**valuation (PRECEDE) model; It has been the first time that this model is adopted for patients with DM in Iran [15]. Among the major constructs in PRECEDE model predisposing, reinforcing and enabling factors (PERF) could be mentioned as an educational diagnosis phase. Therefore, the present study was carried out to survey the effects of (PERF) on self-care behaviors of the patients with DM in the Minoodasht Township, Iran.

## Methods

This study was a quasi-experimental and before after study. The target population of the study was the patients with DM in the healthcare clinics of the township of Minoodasht city, Iran which were selected using convenience sampling method. The sample size considered for statistical formula of this literature review was determined as 78 individuals. The study was carried out after the approved endorsement of the ethical committee at the health department of Iran University of Medical Sciences, and after receiving the official permission from the healthcare center of the Township of Minoodasht. The internal criteria for this study was selected to cover the records of the patients with DM for at least six months and with other types of diabetes, affliction with other diseases, changing the citizenship and the offer to discontinue the study at any time were the external criteria. Also, the patients were assured that the information of the questionnaire and the results were confidential. In the first step the PRECEDE model construct were analyzed and the educational parameters were determined. In the second step, the educational program was compiled and presented on 6 educational sessions. To present the educational content; certain tools were used which included a data projector and different lecturing methods including Questions and Answers, group discussion and different educational materials such as pamphlets, brochures and CDs were employed. After one month, the efficiency of the content of the educational program was determined by using the same questionnaire.

Data gathering was performed using a reliable and valid questionnaire and the questionnaire was set according to different levels in PRECEDE model. In the 4th and 5th levels (educational diagnosis), the possible factors effective on health behavior were identified. These factors included predisposing factors (knowledge, attitude, belief and values), enabling factors and reinforcing factors. The knowledge questions were set as 8 closed questions for participants to answer. Attitude questions were 14 questions according to Likert scale. The first six questions in Likert scale asked about the patients’ attitude and values. Enabling factor questions included 9 questions which asked about the accessibility of the sources and accommodations, the educational classes, the family support, and the skills. Reinforcing factor questions included three questions about the patient positive experiences, the family and the staff encouragement efforts.

Each question was allocated one score for knowledge questions if the answer was correct For attitude questions the Likert scale was employed. Response items were “I completely agree, I agree, I have no idea, I disagree, and I completely disagree”. For any answer, a number from 1 to 5 was considered.

### Statistical analysis

IBM SPSS Statistics 16 for Mac (SPSS Inc., Chicago, Ill) was used for all analyses. Continuous variables are presented as mean (SD) and categorical data as number and percentage. Data were analyzed using paired sample *T*-test and McNemar test

## Results

The mean age of participants was 49 (SD: 3.27) years. Table 1 presents the baseline characteristics of participants.

**Table 1:** **Baseline characteristics of participants**

	**Number**	**Percent**
**Gender**	16	20.5
Male	62	79.5
Female	78	100
Total		
**Educational level**		
Illiterate	15	19.2
Primary school	34	43.6
Secondary and high school	10	12.8
diploma	19	24.4
University level	0	0
Total	78	100
**Marital status**		
Bachelor	0	0
Married	68	87.2
Divorced	0	0
Spouse is dead	10	12.8
Total	78	100
**Number of children**		
2≥	14	17.9
3–5	30	38.5
6≤	34	43.6
Total	78	100
**Income**		
Low	35	44.9
Middle	26	33.3
High	17	21.8
Total	78	100
**BMI**		
<18.5	0	0
18.5–24.9	29	37.2
25–29.9	43	55.1
30–34.9	6	7.7
Total	78	100
**Period of having disease (year)**		
2≥	8	10.3
3–5	20	25.6
6–8	19	24.4
9≤	31	39.7
Total	78	100

The mean scores of knowledge and attitude of DM self-care among female participants (12.14 ± 4.2 and 54 ± 5.6 respectively) was statistically higher than male participant (11.68 ± 2.5 and 51.68 ± 5.5) (p < 0.05).

Enabling factors, which affect self-care behaviors of DM before and after of educational intervention are presented in Table 2. As presented in this table all enabling factors increased significantly after educational intervention. The all reinforcement factors increased significantly after educational intervention (Table 3).

**Table 2:** **Enabling factors affecting in self-care behavior of participants before and after of intervention**

**Enabling factors**			**Before**		**After**		**P- value**
			**Number**	**%**	**Number**	**%**	
Accepting the diet by family		yes	66	84.6	75	96.2	>0.001
		no	12	15.4	3	3.8	
The information source	The friends, family and relative	yes	27	34.6	48	61.5	>0.001
		no	51	65.4	30	38.5	
	Books, brochure, educational film	yes	11	14.1	21	26.9	>0.001
		no	67	85.9	57	73.1	
	Health center staff	yes	61	78.2	72	92.3	>0.001
		no	17	21.8	6	77	
	Newspaper and Magazines	yes	5	6.4	10	12.8	P < 0.05
		no	73	93.6	68	87.2	
	Peer group	yes	16	20.5	23	29.5	>0.001
		no	62	79.5	55	70.5	
Is personnel educational program was affected		yes	63	80.8	73	93.6	>0.001
		no	15	19.2	5	6.4	
Is educational intervention increased the health care skills		yes	34	43.6	56	71.8	>0.001
		no	44	56.4	22	28.2

**Table 3:** **Reinforcing factors affecting in self care behaviors of participants before and after of intervention**

**Reinforce factors**		**Before**		**After**		
		**Number**	**%**	**Number**	**%**	**p-value**
Are you encouraged by health care staff?	Yes	27	34.6	47	60.3	<0.001
	No	51	65.4	31	39.7	
Did your family advice and encouragement in the use of glucose-lowering medication get a diet or doing exercise has had an impact in your disease?	Yes	62	79.5	74	94.9	<0.001
	No	16	20.5	4	5.1	
Did you results through exercise, diet and proper use of medication have earned encourage you to continue in your control?	Yes	58	74.4	67	85.9	<0.05
	No	20	25.6	11	14.1	

Only 49 (62.8%) people performed Insulin control test under the care of a physician before the intervention this figure increased to 58 (74.4%) after the intervention scheme. Also, before the interventional education only 28 (35.9%) people were trained this increased to 49 (62.8%) after receiving the educational intervention. Furthermore; only 26 (33.3%) of the participants attended clinics to receive services and to participate in the educational classes before the implementation of educational intervention, this in turn increased to 41 (52.6%) people after educational intervention.

The mean scores for the knowledge, attitude, and behavior, reinforcement and enabling factors increased significantly (p < 0.001). The mean score of knowledge (12.66), attitude (54.01), behavior (3.98), reinforcement factors (4.46) and enabling factors (1.88) before the intervention changed to 15.32, 56.15, 4.83, 5.29 and 2.41 respectively after the educational intervention.

## Discussion

The findings in the present study showed a significant change in the mean scores of self -care behavior for DM after the intervention (p < 0.001). Appropriate education is fundamental in promoting the knowledge, attitude and behavior of the patients with DM [16,17]. This finding was somewhat concordant with Rezaee et al. study [18].

The findings of the present study also showed that before the intervention some of the patients went on a diet but after the intervention the number of patients that made a dietary change increased significantly. DM belongs to a group of diseases that sufferers have to pay heavy costs for an ongoing treatment so; it is a need for the patients to have a good knowledge of different therapeutic methods, especially nutritional management. In a study by Sharifi Rad et al., it was shown that after educational intervention the mean score for the nutritional behaviors of the patients increased significantly [19].

In the present study, only 62.82% of participants performed Insulin control test under the supervision of a physician before the intervention however, after the study this figure increased to74.35% people. Aghamollaee study shows that there was a significant increase in the scores of the intervention group for personal Insulin control, keeping the diet, weight control, and doing exercise training; but, there was no significant difference observed in the control group [20].

The results in the present study indicated that the mean scores for the knowledge, and attitude factors increased after the educational intervention, which was in line with. In Asghari et al. study [21].

In present study, there was a significant increase in the score of the enabling factors after the intervention and also Mc Nemar test showed a significant difference for enabling factor of deciding to go on a diet by the patient’s families. Overall, most specialists pass on the responsibility of monitoring DM on to the patients and their families. They believe that the patients must take the responsibility of controlling their disease in a manner that is most suitable for their living background and culture [22]. Like all non- diabetic healthy individuals, the patient must play a role in the working place, family, and society [23]. It must be considered that to successfully control their disease, the patients with DM were in need of an enabling program that had been operated since 1989 [24].

In this study there was an increase in the scores of reinforcement factors after the educational intervention and a significant difference in the scores of reinforcement factors such as impact by encouraging staff. In addition, peer support in DM associations is regarded as a common supporting and informative source. Concordant with other studies, receiving education on DM and the complications, from the peer group, family, and the healthcare observer (specially the physician) had a significant effect [25].

The findings in the present study showed that taking advice from the family and receiving their encouragement to use Insulin lowering medication, adapting to go on a diet and taking medication correctly as an incentive factor of controlling the disease. DM is a chronic disease that affects many aspects of the individual’s life, so for the therapeutic treatments, fundamental change in life style choices is necessary. The surrounding social network of the patients, especially by the family [26], dominates these changes. In the study by Heydari et al., it was shown that there was a significant association between family support and Insulin control; the patients who received more support from their family network benefited from a better Insulin control [27]. As a result, because of the significant role of the family, the healthcare staff in the treatment process and educational classes should involve the family. Different studies have shown that the family support is a need for the patients with DM [28].

### Limitation of study

In this study we do not have control group so we cannot compare the result of intervention group with control group.

## Conclusion

Enabling factors such as accepting the diet by family and the source of information and reinforcement factors like encouragement by the health care professional, family advice and encouragement to use the glucose-lowering medication and healthy diet or regular exercise, affects the self-care behavior of the participants.

## Consent

Written informed consent was obtained from the patient for the publication of this report and any accompanying images.

## Endnotes

^a^Any substance to which subjects were sensitive and had mentioned it in the questionnaire.

## Abbreviations

PRECEDE: **P**redisposing, **R**einforcing and **E**nabling **C**onstructs in **E**ducational **D**iagnosis and **E**valuation
DM: Diabetes Mellitus
